# 727. Following WHO priority pathogen list: Carbapenem-Resistant Enterobacterales infections in a hospital in the Dominican Republic

**DOI:** 10.1093/ofid/ofad500.788

**Published:** 2023-11-27

**Authors:** Ricardo Ernesto Hernandez-Landa, Anel E Guzman-Marte, Rita A Rojas-Fermin

**Affiliations:** Universidad Ibero Americana, Santo Domingo, Distrito Nacional, Dominican Republic; Hospital General de la Plaza de la Salud, Santo Domingo, Distrito Nacional, Dominican Republic; Hospital General de la Plaza de la Salud, Santo Domingo, Distrito Nacional, Dominican Republic

## Abstract

**Background:**

Enterobacterales resistant to third generation cephalosporins and carbapenems are included in the WHO priority pathogen list. Evaluating the frequency and carbapenemase production of Carbapenem-Resistant Enterobacterales (CRE) and clinical characteristics of patients in our institution would be a helpful tool in Antimicrobial Stewardship programs.

**Methods:**

A retrospective case-control study was conducted including all CRE patients (42) and a sample of patients with Carbapenem-Susceptible Enterobacterales (CSE), from Jan 2020 to Oct 2022 in a 289-bed tertiary teaching hospital in the Dominican Republic. Demographics, comorbidities, infection source, length of stay (LOS) and antibiotic use were reviewed. Microbiology results were reviewed for resistance rate and carbapenemase production data. Univariable analysis was performed to calculate Odds Ratio (OR), significant correlations were included in multivariable analysis to identify risk factors for CRE infections. P-values ≤ 0.05 were considered statistically significant.

**Figure:**
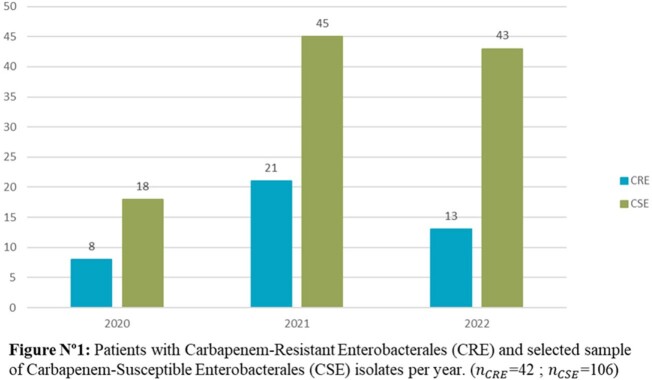

**Results:**

The CRE rate during the 34-month period was 1.5% (45/3022) while 79.5% (36/45) of isolates showed carbapenemase production. The most frequent carbapenemases were KPC 44.4% (20/45) and MBL types 28.9% (13/45); *Klebsiella* spp. (44.4%) along with *Enterobacter cloacae* complex (24.24%) were the most frequent CRE genera. Blood cultures represented the most common source of isolation. Potential risks factors identified in a univariate analysis were LOS ≥15 days, COVID-19 in the previous 6 months, bloodstream infections (BSI), mechanical ventilation, indwelling catheter use and ICU stay, while COVID-19, LOS ≥15 days and BSI resulted as risk factors using logistic regression.

**Figure:**
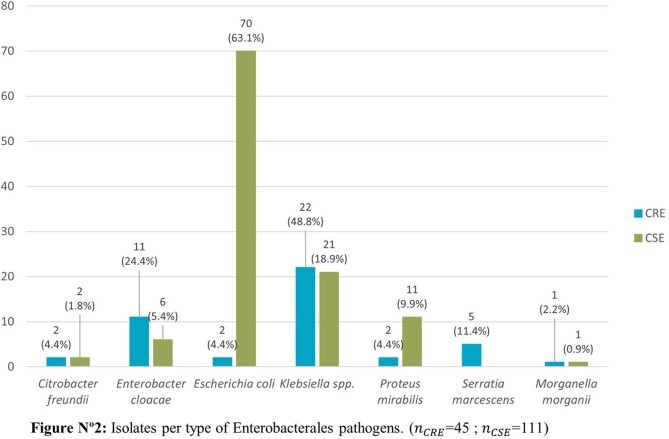

**Figure:**
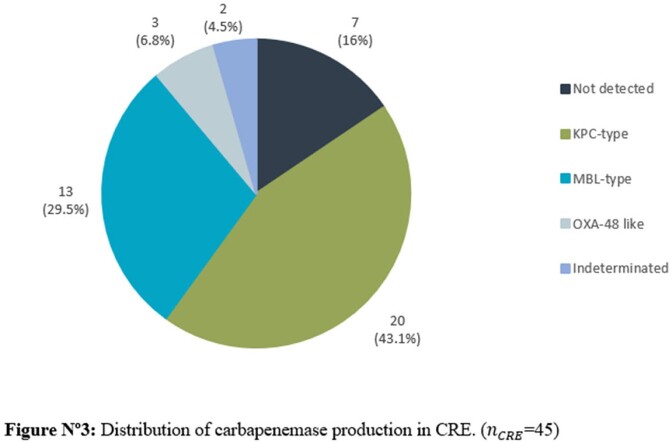

**Figure:**
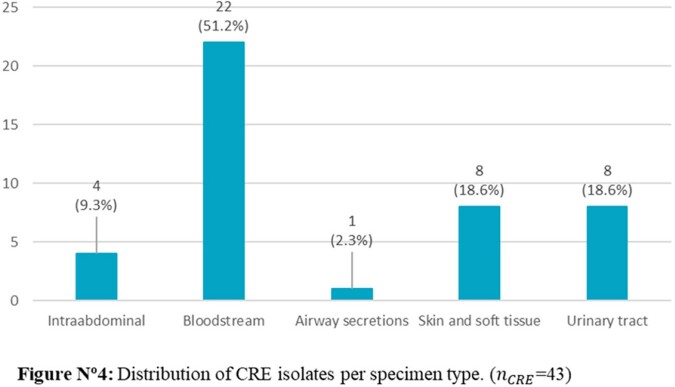

**Conclusion:**

Even though our CRE rates can still be considered low, the incidence of CRE infections has risen since the COVID-19 pandemic. Patients with BSI, LOS ≥15 days and those who have had COVID-19 in the previous 6 months have the highest risk for CRE infections. Since carbapenemase production, mainly KPC and MBL types, complicates patient management and outcome, we emphasize the need of tests that allow us to rapidly detect and identify the type of carbapenemase produced or the presence of simultaneous production.

**Figure:**
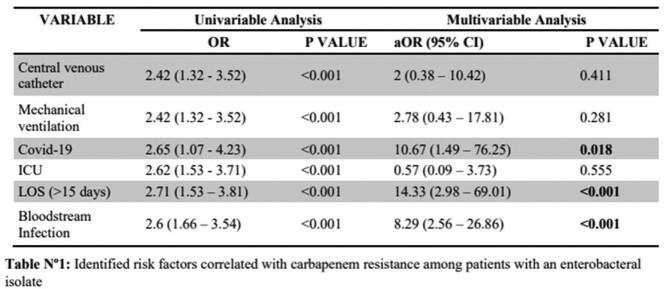

**Disclosures:**

**All Authors**: No reported disclosures

